# Monitoring daily well-being and meaning-making tendencies among adult child working dementia caregivers: validating an experience sampling study protocol

**DOI:** 10.1186/s12877-022-03372-1

**Published:** 2022-08-29

**Authors:** Shuangzhou Chen, Huiying Liu, Vivian W. Q. Lou

**Affiliations:** 1 Department of Social Work and Social Administration, University of Hong Kong, Hong Kong, China; 2 Department of Sociology, Central South University, Changsha, China; 3 Sau Po Centre on Ageing, University of Hong Kong, Hong Kong, China

**Keywords:** Experience sampling method, Protocol design, Feasibility, Meaning-making tendencies, Well-being, Working dementia caregivers

## Abstract

**Background:**

Although the experience sampling method offers advantages for gerontological research, it has seldom been applied to examine well-being and meaning-making tendencies among adult children working caregivers of parents with dementia and thus lacks empirical support for such applications. In response, we aimed to validate the proposed protocol’s participation status, feasibility, usability, and ecological validity.

**Methods:**

For 15 consecutive days, 100 adult child working dementia caregivers participated in our study via web-based assessments on their digital devices. The protocol was first adjusted based on a series of pilot interviews with eight volunteer dementia caregivers. Participants’ compliance and preferred times for activities along with the protocol’s feasibility, usability, and ecological validity were evaluated in a follow-up session with all participants.

**Results:**

The protocol was adjusted in light of recruitment details, user interfaces, the reminder mechanism, and reference time for assessments. The general compliance rate was 93.3%. Preference times for assessments of work (10 a.m. to 3 p.m.), care (6–8 p.m.), and personal activities (7–10 p.m.) were identified. The protocol was generally considered to be feasible and easy to use, and ecological validity analysis indicated that the collected data adequately represented real-world data.

**Conclusions:**

Our study provides empirical evidence to support an innovative protocol and evaluate its implementation so that future studies using it can better investigate the relationship between meaning-making tendencies and well-being among adult child working caregivers for parents with dementia.

## Background

Well-being, including hedonic and social well-being in relation to one’s physical and emotional status, has often been a key outcome investigated in gerontological research, especially its associations with the meaning-making of caregiving experiences in working caregivers of people with dementia (PwD) [1]. However, measuring well-being using conventional approaches has thus far presented several concerns. First, between-person differences have been emphasized, whereas within-person variance has often been overlooked for ideographic purposes during a period of time [2–4], especially regarding the daily status of target populations in studies not using effective designs [4]. Second, in studies using multilevel or panel data, ecological validity has not been prioritized [5], while their accuracy and reliability may have suffered due to recall bias and/or the recency effects [3, 6]. Lacking ecological validity compromises the accuracy of capturing emotional and cognitive experiences and precludes precise reflections of real-life human ecology [7, 8]. For instance, a causal relationship between positive aspects of caregiving experiences and well-being among working dementia caregivers from a cross-sectional perspective is less persuasive than from a longitudinal perspective [1].

Thus, to increase the explanatory power in relation to within-person variances and to improve ecological validity in the measurement of well-being, the experience sampling method (ESM) following the event-contingent approach can be adopted to investigate well-being variances in the occurrence of the same or multiple events over a short time period by collecting daily well-being states simulating real-world data [6, 9, 10]. The ESM, also called “ecological momentary assessment” in some studies, is an intensive longitudinal research method using a structured self-report diary techniques designed to record participants’ momentary thoughts, feelings, behaviors, and perceived environments on multiple occasions intermittently over a short period of time [11–13]. ESM has been commonly used in social psychology and health research, focusing on measuring both subjective (e.g., emotional mood, appraisals, momentary reflections, social interactions) and objective (e.g., psychiatric symptoms, acute health issues, behavioral problems) information in naturalistic settings [13–15]. Based on prior experiences with ESM practices in measuring hedonic and social well-being, our study, by adopting the ESM, can more accurately capture how the variances of caregivers’ well-being are associated with their personal meaning-making tendencies in relation to challenges posed in major aspects of their daily living experiences, including caregiving, work, and personal activities [16, 17]. In the context of dementia caregiving, a multiple-events-contingent ESM approach can be implemented to investigate well-being in daily living experiences of working dementia caregivers. In particular, the event-contingent approach of the ESM involves participatory self-report of occurrences of caregiving, work, and personal life events, in which event-based well-being is recorded simultaneously [12].

Digital devices and platforms are the major venues for conducting assessments following the ESM. Although digital devices have become more popular since the beginning of the twenty-first century [18], the implementation of electronically based designs usually confronts difficulties (e.g., attrition and delayed responses) in comparison with traditional person-to-person or recall-based approaches [3, 6, 19]. Consequently, relevant concerns have been observed in studies using the ESM, including participant non-compliance, the unpredictable availability of participants, and negative user experiences in terms of feasibility and usability [6]. To sustain both the rigor and practicability of proposed ESM-based protocols for studying well-being and meaning-making in relation to experiences with caregiving for PwD, it is crucial to evaluate participants’ compliance and preferred times for activities as well as the method’s feasibility and usability [9, 20].

To date, the application of the ESM in gerontological research has been limited, especially when focusing on the well-being of adult child dementia caregivers in scenarios of care and in terms of their work and personal lives. Adult children worldwide, who represent the largest group of family caregivers for PwD [21], have suffered not only physical problems (e.g., hypertension, diabetes, ischemic heart disease, and stroke) but also psychological burden (e.g., compromised well-being, depressive symptoms, depression, and decreased life satisfaction) [22–24] due to the deteriorating symptoms (e.g., cognitive decline, impaired communication skills, and behavioral problems) observed among PwD [23]. Under such extreme stress, caregivers have an innate need to make sense of their care, work, and personal life experiences as a means to counter their compromised well-being [25–28]. Other than collecting the dates, times, locations, and events of engagement, the ESM designed for research on dementia care records a caregiver’s well-being and meaning-making in order to investigate how their meaning-making tendency in those experiences affects their hedonic and social well-being [6, 12, 29–31].

To ensure the effective implementation of the ESM to monitor the well-being and meaning-making tendencies among adult child working dementia caregivers, the proposed protocol should be adjusted to better address the abovementioned concerns. To that end, our study aims to adjust the overall procedure of the protocol in order to promote well-being among the target caregivers and explore implementation-related outcomes, including the status of participation (i.e., participants’ compliance and preferences for activities) as well as the protocol’s feasibility and usability, and ecological validity.

## Methods

### Design

Our study incorporated the ESM using Qualtrics, an online survey tool, to collect data from digital platforms and operating systems across smartphones, tablets, laptops, and personal computers [32]. End-point devices used among participants included smartphones (95.1%), tablets (3.3%), and personal computers (1.6%) using either the Android operating system or iOS.

We adopted a multiphase approach consisting of the baseline phase, the ESM phase, and the follow-up phase. Prior to an assessment, the target participants were screened by the inclusion criteria. Afterward, each participant received an electronic copy containing an information sheet, a consent form, and instructions for taking the survey using a digital device. At the participant’s request, a phone call was made to provide more detailed and interactive instructions. In the baseline phase, cross-sectional data were collected on personal psycho-socio demographic information, relationships with the PwD, meaning-making, and well-being [33–35]. During the ESM sessions, each participant was separately required to make assessments during their care hours, work hours, and personal hours, respectively. Each assessment was forecasted by a prompt reminding message on the digital device through the pre-installed instant messaging app. Once a participant was reminded and ready to fill the assessment, he or she was provided with the link to the web-based assessment, which included the personal contacts, date and time, location, event of engagement, stress level, coping approaches, and well-being. According to Table 1, personal identification was collected using the phone numbers registered at the baseline. The date and time of each ESM survey being taken were recorded chronologically. The location (among "home", "workplace", "outdoor", and "others to specify") was recorded when a participant clicked the prompt message to the survey. Events of engagement were recorded using one of the following options: "care", "work", "personal activity", and “others to specify”. The stress level perceived during the current activity was scored in the range from 1 (no stress) to 5 (extreme stress). Coping strategies including 8 major coping strategies were recorded as "is currently using”, or “will try later”. Positive and negative affectivities scale including 10 positive affectivities and 10 negative affectivities were scored ranging from 1 (none of this affectivity) to 5 (extreme in this affectivity) [36]. In the end, a schedule for care, work, and personal activities for the next day was provided when a participant has not specified it before or had any emergent change. Based on the pre-scheduled care hours, work hours, and personal hours collected during the baseline phase or the last question of ESM surveys, participants were sent prompt messages and ESM surveys one to three times per day depending on the occurrences of activities among all three types. For instance, participants who work only on weekends, they received two surveys on workdays and three surveys on weekends, in comparison to three times on workdays and two times on weekends for the regular working class. The ESM period continued for 14 consecutive days for each participant. The follow-up phase was conducted right after the 14-day ESM period, during which each participant was again asked about their relationships with the PwD, meaning-making, and well-being during that phase. Upon completion of all phases, the participant was given incentives (i.e., a coupon) acknowledging their contribution to the study. The protocol was designed to follow the principle of voluntary participation, meaning that participants could withdraw from the study at any time at their discretion.

**Table 1:** ESM items

Codes	Items	Description	Details
D0_1	Personal ID	phone number that was registered at the baseline	for internal identification of cases
D0_2	date	date of survey being taken	in the format of YY/MM/DD
D0_3	time	time of survey being taken	in the format of HH:MM:SS
D1	location	the location when the prompt message was received	multiple choices among "home", "workplace", "outdoor", and "others to specify"
D2	events	events of engagement	multiple choices among "care", "work", "personal activity", "others to specify"
D3	stress	perceived level of stress	Likert scale from 1—"no stress" to 5—"extreme stress"
D4	coping	a list of coping strategies	each coping strategy has two options: "is currently using", and "will try later"
D5	PANAS	10 positive and 10 negative affectivities	Likert scale from 1—"none" to 5—"extreme"
D6	schedule	time schedule of care, work, and personal activities in the next day	optional if not previously provided in the baseline

Although recruitment was scheduled to occur from October 2020 to September 2021, the survey was suspended in February for the Lunar New Year holiday and during May and June when COVID broke out in Guangzhou. During the latter period, because most communities were under lockdown for the quarantine purpose, caregivers were restricted from working in their offices and could not bring work back to their homes, either. In addition, outdoor activities were strongly discouraged if not prohibited.

### Eligibility criteria

To be eligible to participate in our study, caregivers needed to (1) be informal, unpaid adult child working caregivers (i.e., ≥ 18 years old) [37]; (2) be residents of Guangzhou (i.e., have local *hukou* or resident status); (3) provide at least 8 h of care per week [21]; (4) be employed for at least 8 h per week [21]; (5) have personal spare time amounting to at least 30 min per day to engage in leisure, recreational, and/or physical activities; and (6) care for a care recipient (CR) medically diagnosed with dementia who had at least one neuropsychiatric behavioral symptoms according to the Neuropsychiatric Inventory Questionnaire (NPI-Q) [38–40] and who was also a resident of Guangzhou (i.e., have local *hukou* or resident status). At the same time, prospective participants were ineligible if they (1) were adult children-in-law providing formal or paid care to a parent living with dementia or were younger than 18 years of age; (2) were not residents of Guangzhou; (3) provided less than 8 h of care per week; (4) were employed less than 8 h per week during the time of the survey or self-employed; (5) possessed less than 30 min per day for leisure, recreational, and/or physical activities; and (6) cared for a CR not diagnosed with dementia or mild cognitive impairment or who presented no neuropsychiatric symptoms according to the NPI-Q (Table 2).

**Table 2:** Inclusion and exclusion criteria

Agent	Items	Inclusion Criteria	Exclusion Criteria
CR			
	Residence	Guangzhou resident	not Guangzhou resident
	Diagnosis	dementia	mild cognitive impairment or not dementia
	Behaviors	NPI ≥ 1	NPI = 0
CG			
	CG type	informal and unpaid caregiver	formal or paid caregiver
	CG role	adult child aged 18 and above	adult–child-in-law or family member other than child; or child younger than 18
	Residence	Guangzhou resident	not Guangzhou resident
	Caregiving	≥ 8 h per week	< 8 h per week
	Employment	≥ 8 h per week	< 8 h per week
	Personal life	≥ 30 min per day for leisure, recreational or physical activities	< 30 min per day for leisure, recreational or physical activities

### Participants

Participants were recruited from community centers and hospitals or via their individual or social connections in Guangzhou. One hundred participants were scheduled to be recruited by the estimation of power analyses for subgroup comparisons, latent class analysis, and hierarchical linear modeling. Initially, a sample size of 70 was considered to be sufficient for conducting an independent *t-test* between subgroups (i.e., with high vs. low well-being) with *p* values less than 0.05 or Cohen’s *d* values greater than 0.8 according to GPower software [41]. Later, latent profile analysis indicated that at least 90 participants would be necessary to infer a profile of meaning-making tendencies predicting one’s level of well-being [42]. Last, a minimum of 50 participants (e.g., 2 groups of 25 participants) was indicated to be necessary to avoid bias in hierarchical linear modeling to estimate the impact of within-person and between-person factors on personal well-being with bootstrapping or simulation-based methods [43–45].

### Adjustment

Prior to all assessments, the potential improvement of the overall procedure was adjusted following pilot interviews. Feedback was gathered from our pilot sample of eight volunteers (mean age was 57.3 years; five women and three men) living with family members with dementia in local communities. Following a mixed-methods approach, the volunteers participated in a brief version of the assessments (i.e., five instead of 14 days) followed by a face-to-face interview [46–48]. Based on the comprehensive feedback, the protocol was adjusted in the following aspects: inclusion and exclusion criteria, feasibility and usability of the web-based user interface and practices, scheduling and reminding system, and the hint for the time duration of the assessments.

### Evaluation and measures

The status of participation (i.e., participants’ compliance and preferences for activities) and the protocol’s feasibility and usability, and ecological validity was examined in a follow-up session after all assessments were completed.

Participants’ compliance and preferences for activities were evaluated after assessments were completed. Although the “force-respond” function was activated in Qualtrics upon the completion of each ESM assessment, that setup may not have guaranteed participants’ a 100% of participation compliance for the online survey. To obtain the target number of assessments deemed sufficient for data analysis, reminders and/or makeup assessments were administered in the case that participants were unavailable for a planned assessment. Rates of compliance were calculated based on the number of assessments completed, divided by the overall number of reminders and/or assessments sent. Preferences for activities afforded insights into the availability of participants in relation to different events.

To measure the protocol’s feasibility and usability, with reference to past experiences with implementing ESM [4] and electronic application on digital devices [49], we incorporated a follow-up scale adopted from past studies [19, 50, 51]. A measurement containing 17 items was designed for a mobile-based setting to evaluate subjective experiences regarding the assessments (e.g., “I filled in the web-based assessment 2 to 3 times a day” and “It was boring to work with the platform”). Responses were given on a 5-point Likert scale ranging from 1 (totally disagree) to 5 (totally agree).

Ecological validity indicated how the collected data represented the real-world physical and social environment [52]. Assessments of the ESM addressed locations and events (e.g., “Where were you when you received the reminder message?” and “What were you doing when you received the reminder message?”). Multiple choices for location included the home, workplace, outdoors (i.e. other than the workplace), and others (i.e., required specification) and for engaged events included care of the CR, care for others (i.e., not the CR), work, physical activities, leisure activities, and others (i.e., required specification).

## Results

### Adjustments

We adjusted the protocol with reference to participants’ feedback in four aspects: the details of their recruitment, the user interface, the reminder mechanism, and referenced duration of the assessment.

First, although the inclusion and exclusion criteria for the protocol were deliberately designed, minor adjustments were needed to avoid certain unexpected situations. Two separate cases particularly raised our attention regarding their recruitment. One participant reported that she usually worked at home while simultaneously caring for her mother; the other mentioned that he always engaged in recreational activities along with his father, including watching television and reading newspapers. Although those two participants nevertheless met the inclusion criteria, they appeared to use their time differently from other participants for two reasons. In the first case, there was no clear-cut approach to determine whether the time used was for a specific activity because work and care were performed simultaneously; in the second, time was reused for two activities (e.g., care and personal activities). The participants remained in the sample, however, because they possessed homogeneous characteristics, meaning-making tendencies, and well-being in relation to the other participants. The recruitment process was therefore adjusted to involve detailed communication prior to assessments in order to prevent similar discrepancies in the future.

Second, the user interface, albeit found to be concise and easy to comprehend, could be customized for different operating systems and devices. All pilot participants (*n* = 8) reported having a clear understanding of the protocol design and questions and found the online platform easy to operate. Participants stated that the assessments were accessible on various digital devices, including smartphones, tablets, laptops, and personal computers, and all reported that the protocol was easier to follow than other paper-based interviews that they had previously completed and more flexible as they shifted between tasks at different venues. Half (*n* = 4) reported completing assessments on a personal computer when at home because the interface looked better on a larger screen. In addition, some (*n* = 2) reported that the version on their smartphones looked dense when they enlarged the font or displayed the assessment horizontally instead of vertically. Therefore, we created a simpler version for individuals who need larger fonts on their smartphones, in which supplementary information and tips are displayed only when they tap or click certain links instead of displaying content directly and thus crowding the interface.

Third, the reminder messages were customized to reliably monitor the punctual completion of assessments. Although reminders followed by phone calls can minimize non-response rates, some participants struggled to be available for timely reminders over the phone while facing a heavy load of tasks. Most (*n* = 5) reported that they could more easily follow the same schedule every day than different ones across the 2-week period. Three participants reported that they had a different schedule on at least one day during the week. The upcoming 2-week schedules of those participants were thus double-checked via a video or audio call prior to their assessments, and a pre-planned schedule for each of those participants was thus recorded for timely reminders for the next 14 days. The schedule for the next day was provided at the end of each day to be confirmed or changed by the participant.

Last, the time duration hint for each assessment was updated as a better reference for participants. Seven of the eight participants indicated that the time required for completing the ESM (i.e., 1–2 min) was more than sufficient compared with the earlier version (i.e., 3–5 min). Most of their assessments were completed in 55 to 70 s once a participant had completed the questions several times and became familiar with them. Moreover, many of them spent 35 min on average on the baseline assessment, compared with 40–60 min previously, and less than 10 min on the follow-up assessment, compared with 15–20 min previously, which repeated the same scales from the baseline and ESM assessments. For adjustment, the estimated time to remind participants with instructions was updated to 30–40 min for the baseline assessment, 1–2 min for each ESM assessment, and 5–10 min for the follow-up assessment. In that way, the estimated duration for each assessment was adjusted on the information page, posters, flyers, and guidelines of assessments when delivered to participants.

### Protocol evaluation

**Fig. 1:**
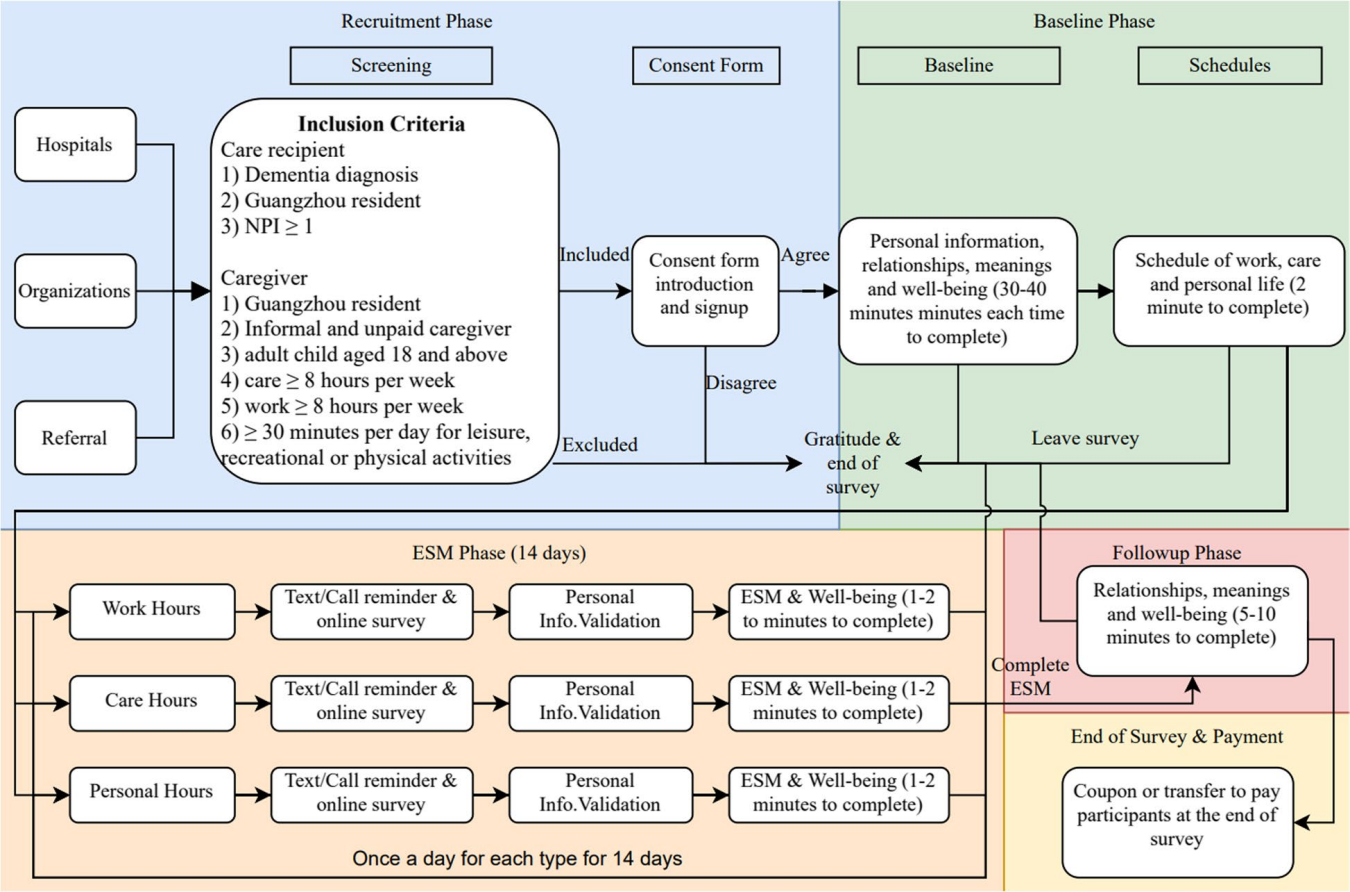
Project flowchart

**Fig. 2:**
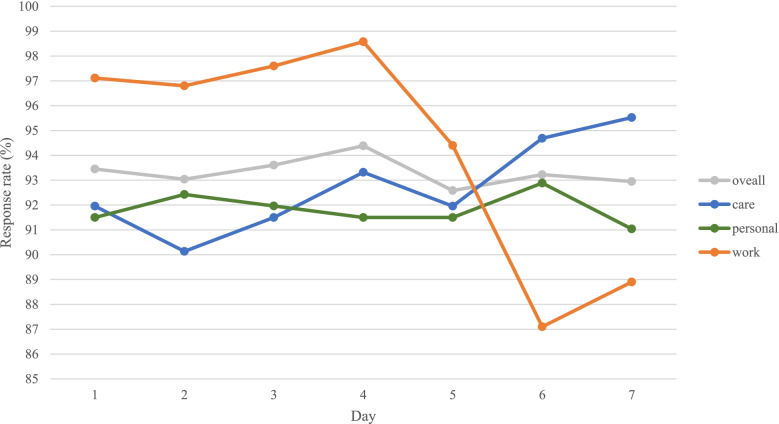
Response rates of scheduled ESM sessions across the week (*n* = 100)

The protocol was evaluated to check participants’ compliance and preferred times for activities and the feasibility, usability, and ecological validity of the protocol. The average time to complete each ESM assessment was 1.1 min (*SD* = 0.54). The sample’s overall response rate (*n* = 100) was 93.3% for the scheduled ESM assessments delivered between 7:00 and 23:59 for the 14-day period. Of the three types of events (i.e., care, work, and personal activities), work had the highest average response rate (96.3%) versus care (92.7%) and personal life (91.8%) but the lowest response rate on weekends (27/22 = 87.1% on Saturdays, 32/36 = 88.9% on Sundays), partly due to the small number of working hours on weekends (Fig. 1). Daily ESM assessments were scheduled based on each individual’s preference at certain time points. As shown in Fig. 2, most responses were made between 18:00 and 22:00; most responses on work were collected between 10:00 and 15:00, and the peak hour was 11:00–11:59 (17.9%). Of all responses about care, most were reported from 18:00 to 20:00, and the peak hour was 19:00–19:59 (25.2%). Of all responses on personal activities, most were reported between 19:00 and 22:00, with the peak during 21:00–21:59 (37.1%).

According to the evaluation of feasibility and usability shown in Table 3, participants perceived the web-based ESM survey platform as “easy to use” (*M* = 3.91, *SD* = 1.02), “fun to work with” (*M* = 3.21, *SD* = 1.45), and “easy to understand” (*M* = 4.17, *SD* = 0.96). Most participants agreed that they carried a smartphone every day (*M* = 4.56, *SD* = 1.31) and that doing so was easy (*M* = 4.33, *SD* = 1.01). Using Qualtrics as the web-based survey platform, participants agreed that the ESM assessments were highly accessible on digital devices and generally “worked well” (*M* = 4.75, *SD* = 2.81). Regarding the frequency of the online assessments, most participants engaged with the assessments 2 to 3 times per day (*M* = 4.68, *SD* = 1.31) during the 14-d period (*M* = 4.88, *SD* = 0.86). Prompts for assessments were perceived as “reasonable” (*M* = 3.68, *SD* = 0.38) more than “annoying” (*M* = 2.32, *SD* = 1.84), as well as “well-displayed” (*M* = 4.11, *SD* = 1.72) and “easy to complete” (*M* = 4.41, *SD* = 2.30) on a smartphone. Even so, the assessments were reported to be time-consuming (*M* = 3.55, *SD* = 1.25) and, to a moderate degree, “an interruption of daily activities” (*M* = 3.09, *SD* = 2.61) despite being “understood” (*M* = 3.80, *SD* = 2.22).

**Table 3:** Feasibility and usability measures (*n* = 100)

Item		M ^1^ (SD)	% ^2^
1	The web-based survey platform is easy to use	3.91 (1.02)	64.4
2	It is easy to carry the smartphone with me	4.33 (1.01)	57.4
3	I carried my smartphone with me every day	4.56 (1.31)	67.3
4	After the researcher’s explanation I understood how the web-based survey platform would work	4.17 (0.96)	64.4
5	It was fun to work with the platform	3.21 (1.45)	69.3
6	It was boring to work with the platform	2.89 (1.08)	42.6
7	The web-based survey platform worked well	4.75 (2.81)	46.5
8	I experienced the prompts as reasonable	3.68 (0.38)	44.6
9	The number of prompts was annoying	2.32 (1.84)	49.5
10	I filled in the web-based assessment for 14 consecutive days	4.88 (0.86)	87.1
11	I filled in the web-based assessment 2 to 3 times a day	4.68 (1.31)	91.1
12	It was easy to fill in the web-based assessment on my smartphone	4.41 (2.30)	49.5
13	The questions were well-displayed on my smartphone	4.11 (1.72)	44.6
14	Filling in one web-based assessment was an interruption of my daily activities	3.09 (2.61)	41.6
15	Filling in one web-based assessment took too long	1.79 (1.01)	71.3
16	The study took too long	3.54 (1.25)	79.2
17	I understood the questions that were asked	3.80 (2.22)	86.1

^1^ *Scores were based on a 5-point Likert scale (ranging from 1—totally disagree to 5—totally agree)*

^2^ *Percentage of scores above the mean*

Ecological validity analyses (Table 4) indicated that responses covered a decent range of events and locations representing the real ecological environment. Of all categories of events, care was the major event (42.0%), followed by work (31.8%) and personal activities (26.2%). Caregiving was more often provided at home (86.3%) or in other places (11.8%), most often in relatives’ homes, hospitals, or community centers. Caregivers’ mostly performed their work as employees at a workplace (79.2%), albeit some more often at home (11.1%) due to flexible and/or part-time regimes during the COVID-19 pandemic. In addition, the validities of relevant scales have been evaluated using factorial analysis, ranging from 0.714 (Occupational Stress Inventory) to 0.922 (Unidimensional Relationship Closeness Scale). The internal reliabilities of these scales ranged from 0.74 (Occupational Stress Inventory) to 0.91 (Unidimensional Relationship Closeness Scale).

**Table 4:** Ecological validity (*n* = 100)

Events	location ^1^				Total ^2^
	Home	Work	Outdoor	Others	
Care	1379 (86.3%)	28 (1.8%)	1 (0.1%)	189 (11.8%)	1597 (42.0%)
Work	134 (11.1%)	958 (79.2%)	38 (3.1%)	79 (6.5%)	1209 (31.8%)
Personal	869 (87.4%)	2 (0.2%)	89 (9.0%)	34 (3.4%)	994 (26.2%)
Total	1513 (62.7%)	986 (26.0%)	39 (3.4%)	268 (7.9%)	3800 (100%)

*Note*: ^1^ *all row percentages*

^2^ *all column percentages*

### Implementation procedure

**Fig. 3:**
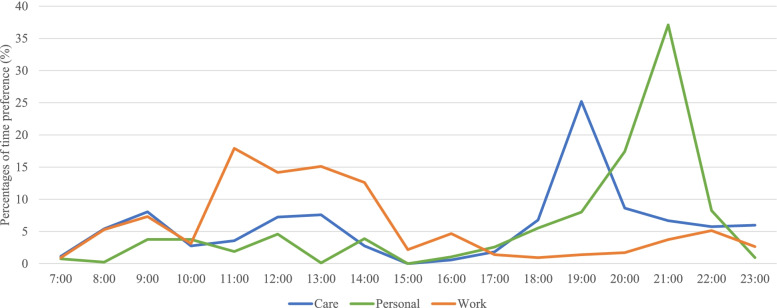
Time preference throughout the day (*n* = 100)

The implementation procedure of the protocol consisted of three phases: the baseline phase, the ESM phase, and the follow-up phase (Fig. 3).

The baseline phase of the survey needed to be completed on the weekend or whenever participants were free—ideally, one day before the beginning of the ESM phase. During the baseline phase, participants were asked to report their CRs’ daily living capacities (i.e., activities of daily living and instrumental activities of daily living) and symptoms (i.e., severity level on the NPI-Q); their own demographic information (e.g., gender, age, marital status, level of education, religious belief, duration of caregiving in months, living arrangement, caregiving role, economic condition, average hours per day spent on care, work and personal life, and time taken to adapt to caregiving role); their overall mental status (i.e., disturbances on the NPI, Zarit Burden Inventory, Positive and Negative Affect Schedule (PANAS)); hedonic well-being and social well-being (SWB) [53–58]; and the dyadic relationship between themselves as caregivers and their CRs in the past and during caregiving (i.e., with the Unidimensional Relationship Closeness Scale) [59]. Meaning-making of care, work, and personal life, on the Positive Aspects of Caregiving, Work and Meaning Inventory, and Meaning in Life Questionnaire, were reported at baseline as well [60–62]. In addition, personal schedules for care, work, and personal activities from 6:00 to 23:59 for the next day (or more days during the next two weeks, if could be provided) were collected prior to the beginning of the next phase for a timely and accurate assessment based on the ESM.

Next, the ESM phase started on a Monday and continued for 14 consecutive days. Throughout each day, participants were reminded by prompts to report each event in their care, work, and personal life and its location, as well as their coping strategies and perceived hedonic and social well-being (i.e., PANAS and SWB) [6]. Throughout the 14-day period, reminders were sent to participants based on their preferred time points reported in advance. An additional prompt allowed participants to provide the schedule for the next day if they had not previously provided any future schedule or needed to accommodate any short-notice changes. Prompts included a reminder function sent to a social networking app accompanied by a link to the online web-based assessment. The reminder in the app displayed “Accomplished” once the participant clicked the reminder that directed them to the assessment. Qualtrics was also set up to email us detailed content whenever a participant finished an assessment. In that way, we were able to monitor the process and the completeness of every assessment at any time.

The follow-up phase, beginning directly after the end of the ESM phase, was designed to assess participants’ overall well-being (i.e., PANAS and SWB), meaning-making of care (i.e., PAC), life (i.e., MLQ), and work (i.e., WAMI, reported on a workday). Any participant who missed more than one-fourth (≥ 10) of the ESM assessments were excluded from the sample [63].

### Statistical analysis

R3.6.0 and SPSS 24.0 were used for analyses and to create tables in APA Style, while Microsoft Excel was used to generate figures in APA Style. To begin, multilevel analyses were conducted to compare caregivers according to age group, gender, level of education, the severity of their CR’s dementia, caregiving conditions, and work hours. Second, latent profile analysis was used to analyze characteristics and well-being across profiles of caregivers who had different tendencies to make meaning of events in their care, work, and personal life. Third, hierarchical linear modeling was used to examine the trajectory of well-being influenced by variances within an individual (i.e., Level 1) and personal characteristics (i.e., Level 2), as well as in the profiles of caregivers with different meaning-making tendencies (i.e., Level 3). Those participants who have ¼ missing data were also excluded from the analyses. Sporadic missing data was processed using mean-, regression-, or multiple-imputation techniques depending on the properties of the variables [64].

### Data management and confidentiality

The risk of data was kept at a low level as only three copies were made to store the data. The laptop, external hard drive, and server were protected by passcodes. Collected data were archived with version control eliminating sensitive personal information while using the unique identifier for reference to the source in case of retrieving missing data. The master list of identifiers corresponding to participants’ personal information was stored in a designated electronic device with passcodes. Other personal information such as the address, date of birth, and phone numbers irrelevant to the study were not collected nor registered unless they were relevant to any research questions in this project.

The risk of leakage of data was low as all data were passcode-protected. Only authorized researchers and staff had the access right. The data were stored in a passcode-protected laptop, external hard drive, and backup server in the laboratory. Data were backed up monthly in the laboratory server with copies at three different devices. Data were planned to be deleted five years after this study completes.

### Ethical consideration and dissemination

Following the integrity and the guideline of research ethics and policy, this study was registered and approved on October 8, 2020, by the Human Research Ethics Committee at the University of Hong Kong (EA200080) to ensure ethical clearance for research involving human participants.

Participants were required to give written informed consent before the survey. Data anonymity and confidentiality were ensured during their participation. The collected data of this study were planned to yield a series of manuscripts that aim for presentation and publication in a timely fashion once the data is collected and analyzed. The available findings of manuscripts during the stages of the project can be disseminated to the scientific community, such as public media, the annual academic conferences, and submitted to social science journals for publication.

## Discussion

To our best knowledge, our study marks the first attempt to investigate well-being and meaning-making tendencies among adult child working dementia caregivers. Our aim was to perfect the overall procedure of the proposed protocol so that future studies using the design can be developed to investigate the well-being among these caregivers and examine participants’ compliance and preferred times for activities, as well as the protocol’s feasibility and usability, and ecological validity.

The protocol’s adjustment has improved its capacity to investigate the relationship between meaning-making tendencies and well-being among working dementia caregivers, in terms of generalizability, usability, and feasibility. Above all, the updated recruitment approach not only made data collection more precise but also increased the generalizability of the results by increasing the representativeness of the sampled population. The recruitment and analysis with 100 participants were acceptable, for it allowed decent statistical power (i.e., *p* < 0.05 and Cohen’s *d* > 0.8) for group comparisons and hierarchical linear modeling [41]. The survey was assessed to be equipped with sufficient guidelines, online supervision and tutorials, and customized responses as well as validated to maximally reduce experimental errors (e.g., unforced errors, misspellings, non-response errors, and experimental biases) [65]. With a customized design and display on various devices, the updated interface was more user-friendly and easier to interact with on different devices. Such flexibility in user interface and improvement in user-friendliness avoid the kind of incompatibility often reported by users of built-in mobile apps, which are also costly if they extend their compatibility across different operating systems [66]. ESM assessments following customized, timely reminders and daily routines according to the next day’s schedule prevented unresponsiveness from participants. Even though the updated reference times did not affect the actual times that participants spent on the assessments, we wanted participants to be as informed as possible and did not want to present misleading information. Overall, the adjusted protocol demonstrated desirable validity and reliability in exploring the relationship between meaning-making tendencies and well-being.

The evaluation of the adjusted protocol clarifies participants’ compliance and preferred times for activities, as well as the satisfactory feasibility, usability, and ecological validity of the protocol. With a reminder mechanism developed with a mixed-methods approach, the protocol’s response rate of 93% exceeds that of protocols solely using one pop-up reminder for each assessment [67, 68]. The summarized preferred times for activities inform the effective implementation of the ESM assessments and how they ensured reliable data collection and prevented delays or truancy in responses. Given its enhanced feasibility, the protocol facilitates daily ESM assessments, each of which takes only 1–2 min to complete, which neither interrupts daily activities nor brings undue mental burden to participants. The updated protocol thus seemed effective and easy to use on a daily basis even when the participants had to complete a variety of other tasks at different time points throughout the day. Our findings confirm that the ESM design has desirable feasibility and usability in measuring the daily conditions of dementia caregivers [9, 69]. However, our protocol does not come across the issues associated with digital ignorance or digital divide due to homogeneous younger participants in their 40 s [10]. On top of that, decent ecological validity was reflected in several aspects. For one, the protocol fully captured the real life of a dementia caregiver who was also employed in another occupation regarding their daily engaged events and locations. For another, the 15-day design (i.e., one day at baseline and 14 days during the ESM and follow-up phases), compared with a cross-sectional design, was able to better depict the variances and patterns of a caregiver’s well-being and how caregivers cope with daily difficulties in a more timely and accurate manner [6]. Moreover, unlike traditional ESM-designed studies focusing on a single event [6, 12, 29, 63], the protocol is proved to successfully evaluate three independent events in a target population from a dynamic, trajectorial perspective, one that best outlines a more well-rounded life experience of such caregivers. The repeated measure consisting of ESM-related questions about making meaning in the daily care, work, and personal activities among dementia caregivers over the 2-week period can thus empower the caregivers to more fully consider the significance of meaning-making to the well-being.

Given the rigor and feasibility demonstrated in the design of this protocol, a few things can still be perfected. Although participants in our study could all have access to digital devices (e.g., smartphones), such digital devices may remain unavailable in certain communities or among older age groups who do not heavily rely on electronic commerce or social media networks, for example, due to low digital literacy or limited Internet access [70]. In response, smartphones with data plans can be lent to participants for surveys in the case that they have no digital devices or Internet access [71]. Moreover, people using technological devices in ESM-oriented research may confront difficulties if their knowledge about digital devices is not up-to-date [72, 73]. Therefore, a detailed tutorial or pre-session training via video conference or audio call should be provided to individuals who lack digital literacy. Such an arrangement can also build rapport with participants and minimize the occurrence of unpredictable situations that each participant may confront on a daily basis. The design used for this specific protocol is slightly different from common ESM approaches. Since we investigate three different types of activities of working dementia caregivers, the efficient arrangement should be designed to avoid overlapping or mismatched assessments in corresponding time periods. Therefore, such a multiple-events-contingent ESM design can be further validated in future studies.

In sum, to our knowledge, this study is the first to have investigated the relationship between meaning-making tendencies and well-being among adult–child working dementia caregivers. Our study empirically evaluated the implementation-related outcomes of the protocol and thus offered insights that can inform the future application of the protocol on digital devices in gerontological research. For instance, future studies may refer to the protocol to probe trends among caregivers of PwD in greater depth and in terms of their CRs’ different subcategories of dementia (e.g., vascular dementia, Alzheimer’s disease, Lewy body dementia, and frontotemporal dementia) or phases of dementia (e.g., early and late phases). Interventions and support services using effective meaning-oriented approaches can be more useful once specific subtypes of PwD and their caregivers are identified and, in turn, can better benefit specific PwD populations as a result of future empirical studies and improved clinical practices [74, 75]. The protocol can moreover serve as a preliminary reference tool for cultural comparisons designed to investigate cultural impacts on caregivers’ meaning-making tendencies and perceived well-being among a broader sample of diverse cultures and/or socioeconomic contexts.

## Data Availability

The datasets used and/or analyzed during the current study are available for the upcoming doctoral thesis and a few more manuscripts not yet published. The data could be provided from the corresponding author on reasonable request afterward.
